# Extraction Methods and Sedative–Hypnotic Effects of Total Flavonoids from *Ziziphus jujuba* Mesocarp

**DOI:** 10.3390/ph18091272

**Published:** 2025-08-26

**Authors:** Jie Li, Baojian Li, Xinbo Shi, Yuangui Yang, Zhongxing Song

**Affiliations:** 1 Shaanxi Collaborative Innovation Center of Chinese Medicine Resources Industrialization, Shaanxi University of Chinese Medicine, Xianyang 712046, China; lijie9596@sina.com (J.L.); 1501026@sntcm.edu.cn (X.S.); 2 School of Pharmacy, Xinjiang Second Medical College, Karamay 834000, China; lbj9591@163.com; 3 School of Pharmacy, Xinjiang Medical University, Urumqi 830017, China

**Keywords:** *Ziziphus jujuba* mesocarp, total flavonoid, extraction process, sedative-hypnotic effects

## Abstract

**Background/Objectives**: As a non-medicinal part resource of *Ziziphus jujuba*, this study focuses on the total flavonoids from *Ziziphus jujuba* mesocarp (TFZJM), aiming to optimize the extraction process and explore its sedative and hypnotic effects. **Methods**: The extraction process of TFZJM was optimized by using single-factor experiments and the Box-Behnken response surface design method. The material basis of TFZJM was analyzed using Ultra-Performance Liquid Chromatography-Quadrupole-Time of Flight-Mass Spectrometry (UPLC-Q-TOF-MS). The mouse insomnia model was induced by intraperitoneal injection of PCPA, and the effects of TFZJM on this model and its potential mechanism were evaluated using multiple methods, such as sleep enhancement induced by pentobarbital sodium, HE staining of tissue sections, ELISA, RT-PCR, WB, and serum metabolomics. **Results**: The results showed that by optimizing the extraction conditions, a solid-liquid ratio (SLR) of 1:25 g·mL^−1^, ethanol concentration of 60%, extraction time of 60 min, and extraction rate of 1.98% were achieved. The common chemical basis of the 10 flavonoid components was identified using UPLC-Q-TOF-MS analysis. Compared with the model group, the high-dose TFZJM (TFZJM-H) group had the most significant effect, followed by the medium-dose (TFZJM-M) and low-dose (TFZJM-L) groups. **Conclusions**: Metabolomic analysis revealed that TFZJM regulates pathways related to the metabolism of phenylalanine, tyrosine, cytochrome P450, and alanine. This lays the foundation for further exploration of the active substances and mechanisms of action of TFZJM in sedation and hypnosis.

## 1. Introduction

Insomnia is a prevalent sleep disorder characterized by a range of physical, psychological, and behavioral clinical manifestations. Prolonged insomnia contributes to depression, exacerbates fatigue, impairs cognitive function, and compromises immune responses [1,2,3]. Contemporary studies have highlighted that neurotransmitter balance directly influences sleep quality [4,5]. Neurotransmitters, which mediate communication between neurons, are integral to brain function, particularly in sleep regulation. Their release and modulation are closely linked to sleep quality [6,7]. Gamma-aminobutyric acid (GABA), an inhibitory neurotransmitter, diminishes neuronal excitability through its interaction with GABA receptors, inducing sedative and hypnotic effects. This is achieved by upregulating GABA_A_R_α_1 expression, which directly influences GABA receptor availability [8,9,10]. Similarly, 5-hydroxytryptamine (5-HT), a neurotransmitter integral to the regulation of mood, sleep, and appetite, is widely recognized for its therapeutic potential in addressing insomnia, and increasing 5-HT levels in the brain is considered a beneficial approach [11,12,13]. Furthermore, research indicates that insomnia is linked to decreased levels of brain-derived neurotrophic factor (BDNF). Enhancing BDNF levels has been shown to improve sleep quality, underscoring BDNF’s role in supporting cognitive function and restoring healthy sleep patterns [14,15].

*Ziziphus jujuba* [*Ziziphus jujuba* Mill. *var. spinosa* (Bunge) Hu ex H. F. Chou], a woody plant of the Rhamnaceae family, is mainly distributed as a food in Asia, Europe, and parts of the Americas. In China, it is mainly distributed in Shaanxi, Shanxi, Hebei, Shandong, and other provinces, with an annual production exceeding 30,000 tons [16]. Jujuba seeds are widely consumed as a fruit due to their sour and sweet taste, and their seeds, known as jujuba kernels, are the preferred medicine for insomnia and are widely used in clinical prescriptions for insomnia. During the processing of jujuba kernels, the mesocarp is discarded and not effectively utilized, resulting in waste of resources and damage to the ecological environment [17,18]. Therefore, it is urgent to further study and effectively use *Ziziphus jujuba* mesocarp.

In Zhang Zhong-jing’s Essentials from the Golden Cabinet (Han Dynasty), it was stated that “deficiency-consumption and vexation cause insomnia, and *Ziziphus jujuba* Decoction is used as treatment,” highlighting its therapeutic potential in insomnia. Further, the Newly Revised Materia Medica emphasizes the efficacy of *Ziziphus jujuba* mesocarp in addressing insomnia, reflecting its historical role as a medicinal substance. *Ziziphus jujuba* seed, listed in the Chinese Pharmacopoeia, contains bioactive components such as flavonoids, saponins, alkaloids, and fatty acids, known for their sedative, antioxidant, anti-inflammatory, and cardioprotective properties [19]. The mesocarp of *Ziziphus jujuba* also contains flavonoids, polysaccharides, alkaloids, and other compounds with sleep-enhancing, hepatoprotective, and anti-tumor activities [20]. Flavonoids are key active components of *Ziziphus jujuba* seeds, and modern pharmacological research has demonstrated their efficacy in improving sleep quality [21]. Research has found that the total flavonoids of jujuba seeds and some monomer compounds (spinotropin and doxanthin) mainly exert sedative and sleep-inducing pharmacological effects by regulating three neurotransmitters: 5-HT, norepinephrine (NE), and GABA [22,23,24]. Zhang et al. found that spinoxin can significantly reduce the expression of proto-oncogene protein (c-Fos) in the lateral hypothalamus (LHA) and locus coeruleus (LC) of the brain, indicating that spinoxin may exert its sedative-hypnotic biological activity by downregulating the expression of c-Fos and thereby reducing neuronal discharge [25]. Research has found that TFZJM can downregulate glutamic acid (GLU) and upregulate BDNF, exerting its pharmacological activity of relieving depression and calming the mind. In addition, Jiang et al. found that the sedative and sleep-inducing abilities of flavonoids in jujube are weaker than those of jujube seed saponins, and jujube polysaccharides have no sedative or sleep-inducing effects [26]. Clinically, *Ziziphus jujuba* seeds have been widely used to treat various types of insomnia, with studies primarily focusing on the sedative and hypnotic mechanisms of their flavones. However, limited attention has been given to the comprehensive clarification of the pharmacodynamic substances and potential mechanisms of TFZJM for sedative and hypnotic effects.

The aim of this study was to optimize the extraction and purification processes of TFZJM and further analyze the pharmacodynamic basis and mechanism of the sedative and hypnotic effects of the TFZJM extract, laying a foundation for the resource development and utilization of non-pharmaceutical parts of Jujuba.

## 2. Results

### 2.1. Optimization of TFZJM Extraction Conditions by Single-Factor Experiment

**Figure 1:**
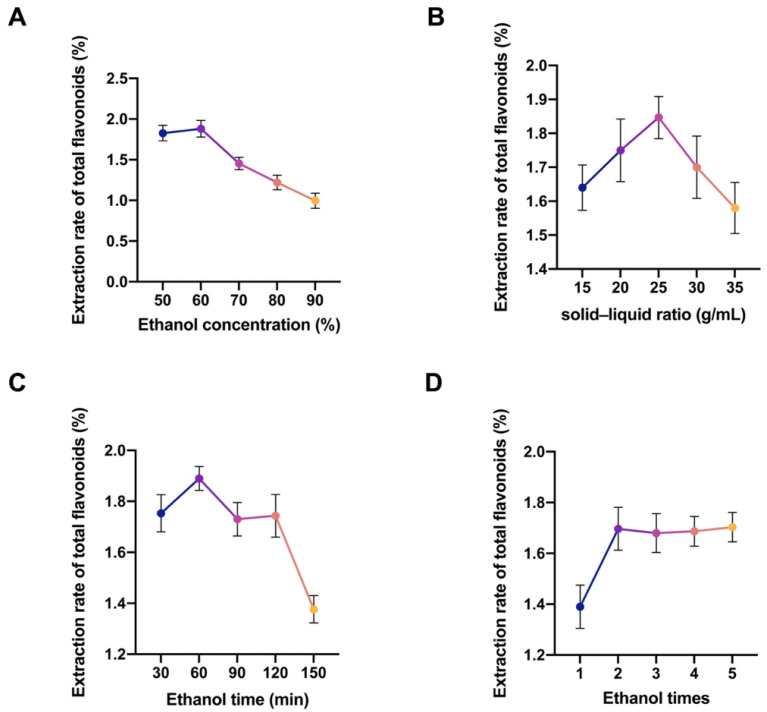
Single-factor investigation of TFZJM extraction conditions. (**A**) Single-factor investigation of ethanol concentration; (**B**) single-factor investigation of SLR; (**C**) single-factor investigation of extraction time; (**D**) single-factor investigation of extraction times. The different colors represent the extraction rates of total flavonoids with different ethanol concentrations, different SLR, and different extraction times.

The impact of ethanol concentration on the extraction of TFZJM is illustrated in Figure 1A. The extraction efficiency initially increased with increasing ethanol concentration, peaking at 60% before declining as the concentration continued to increase. Therefore, 60% ethanol was selected for further experiments. The effect of the SLR on extraction efficiency, with optimal extraction occurring at a ratio of 1:25 g·mL^−1^, is shown in Figure 1B. Beyond this point, the efficiency decreased, confirming a ratio of 1:25 g·mL^−1^ as ideal for subsequent tests. The extraction time significantly influenced extraction, with the maximum extraction achieved at 60 min, which was chosen for further analysis (Figure 1C). Increasing the number of extractions enhanced the extraction of TFZJM, which stabilized after two extractions (Figure 1D). Therefore, two extraction cycles were selected for the response-surface experiment.

### 2.2. Statistical Analysis and Model Fitting

A response surface experimental design was used, and the results are summarized in Table 1. Regression fitting analysis was performed using Design Expert 13, where extraction time (A), ethanol concentration (B), and SLR (C) were considered independent variables, and total flavonoid extraction efficiency (Y) was the response variable. The resulting multivariate quadratic equation was expressed as follows: Y% = 1.92 − 0.0050A + 0.0325B + 0.0275C − 0.0075AB − 0.0875AC + 0.0175BC − 0.3488A2 − 0.1938B2 − 0.1387C2.

The statistical analysis of the Analysis of Variance (ANOVA) is presented in Table 2. The *p*-value of the model was less than 0.0001, indicating that the model had a significant effect on the extraction rate of TFZJM. The F and *p* values of the lack-of-fit term are 0.0556 and 0.9805, respectively, indicating that the lack-of-fit value is not significant. This confirms that the model has a good fit with a small error, suggesting that the model is valid. The analysis of variance shows that the first-order terms B and C and the second-order terms A2, B2, and C2 all exhibit significant levels. In the regression model, R^2^ is 0.9929 and the adjusted R^2^_adj_ is 0.9837, indicating that the model has a high goodness of fit and a high correlation between the predicted values and the measured values. The coefficient of variation (CV) is 1.87, indicating that the model has high precision and reliability. In conclusion, the established model of the extraction rate of TFZJM has high accuracy and credibility and can be used for predictive analysis of the extraction amount of TFZJM. The three first-order terms (A, B, C), the three second-order terms (AB, AC, BC), and A2, B2, and C2 all have a relevant influence on the extraction rate of TFZJM, indicating that the influence of the three factors on the extraction rate of TFZJM is not a simple linear function relationship. The F values of the three factors are in the order B > C > A, indicating that the order of the influence of each factor on the extraction rate of TFZJM is ethanol concentration > SLR > time. Extraction time and ethanol concentration, SLR (AB, AC), and ethanol concentration and SLR (BC) also significantly influence the extraction rate of TFZJM.

### 2.3. Response Surface Analysis of TFZJM Extraction Process

**Figure 2:**
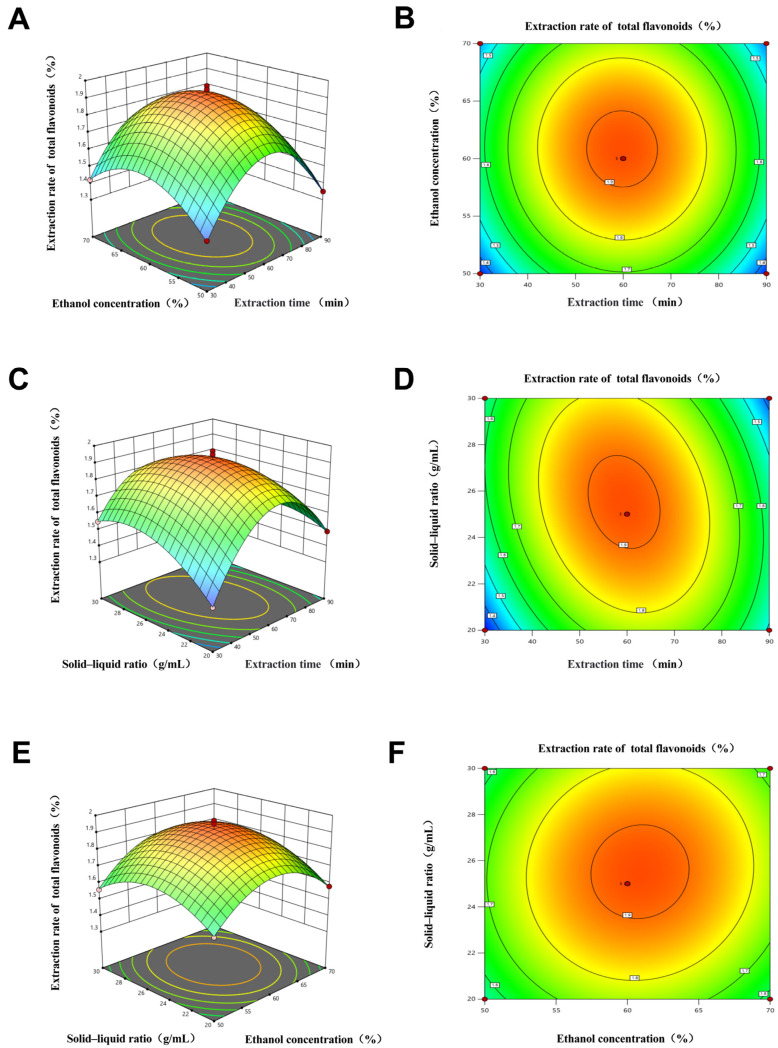
Results of the interaction of various extraction factors of TFZJM. (**A**) Response surface diagram of ethanol concentration and extraction time to total flavonoid extraction rate; (**B**) Ethanol concentration and extraction time on total flavonoid extraction rate contour map; (**C**) Solid–liquid ratio and extraction time to total flavonoid extraction rate response surface; (**D**) Solid–liquid ratio and extraction time to total flavonoid extraction rate contour map; (**E**) Solid–liquid ratio and ethanol concentration on the response surface of total flavonoid extraction rate; (**F**) Solid–liquid ratio and ethanol concentration on total flavonoid extraction rate contour map. Note: The red dot in the center of the graph represents the optimal condition where two conditions intersect, and the red dots around it represent the range of the interaction conditions.

To enhance the understanding of the interactions among test variables, response surface and contour plots (Figure 2) were generated to illustrate the effects of the extraction time, ethanol concentration, and SLR on the extraction rate of TFZJM. The response surfaces for these interactions exhibited a downward opening, indicating that the extraction rate of TFZJM initially increased before subsequently decreasing as A, B, and C were elevated. The interaction effects of the ethanol concentration and extraction time on the TFZJM extraction are depicted in Figure 2A,B. The flavonoid extraction rate increased markedly as the ethanol concentration approached 60% and the extraction time reached 60 min, after which it declined. The influence of the SLR and extraction time at a fixed ethanol concentration of 60% is illustrated in Figure 2C,D, with the highest extraction efficiency achieved at an SLR of 1:25 g·mL^−1^ and an extraction time of 60 min. Similarly, the combined effects of SLR and ethanol concentration on flavonoid extraction at an extraction duration of 60 min are demonstrated in Figure 2E,F, indicating that the optimal extraction efficiency occurred when the SLR was 1:25 g·mL^−1^ and the ethanol concentration was 60%. The response surface model predicts the optimal extraction process as follows: extraction time of 59.334 min, ethanol concentration of 60.87 %, SLR of 25.562:1 g·mL^−1^, and total flavonoid extraction rate of 1.923%. Considering the operational errors, the optimized experimental conditions were as follows: extraction time, 60 min; ethanol concentration, 60 %; and SLR, 25:1 g·mL^−1^. Three verification experiments were conducted under these conditions, and the calculated total flavonoid extraction rate was 1.983 ± 0.012%, with a small error from the predicted value, further verifying the reliability and stability of the model.

### 2.4. Results of TFZJM Purification Process

Adsorption and Desorption Rates of Various Macroporous Resins.

**Figure 3:**
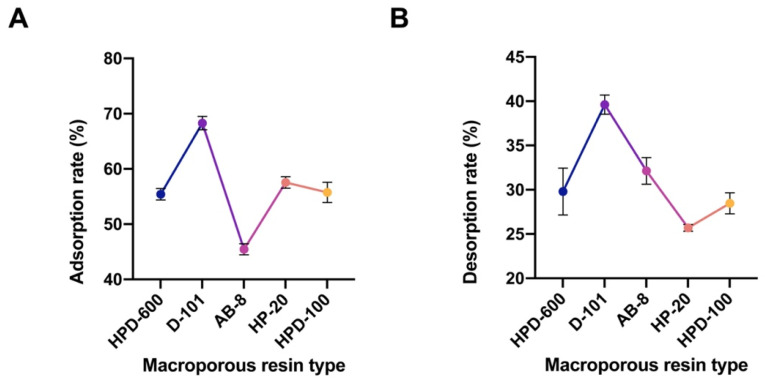
Screening of macroporous resin types. (**A**) Adsorption rate; (**B**) Desorption rate. Note: Blue represents the adsorption and desorption rates of the HPD-600 type; purple represents the adsorption and desorption rates of the D-101 type; pink represents the adsorption and desorption rates of the AB-8 type; orange represents the adsorption and desorption rates of the HP-20 type; yellow represents the adsorption and desorption rates of the HPD-100 type.

Five types of macroporous resins—HPD-600, HPD-100, D-101, HP-20, and AB-8—were evaluated based on their adsorption and desorption rates. These parameters were used to select the optimal resin model. The analysis revealed that D-101 resin exhibited superior adsorption and desorption rates, leading to its selection as the most suitable resin for the adsorption process (Figure 3).

### 2.5. Effect of TFZJM Extract on Adsorption Rate at Different pH Values

**Figure 4:**
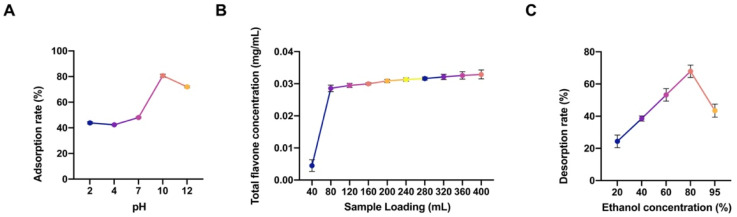
Results of the investigation of the TFZJM purification process. (**A**) Adsorption rate under different pH conditions, (**B**) maximum adsorption capacity, and (**C**) desorption rates of different ethanol concentrations. Note: Different colors represent adsorption rates under different pH conditions; Different adsorption capacities; And the desorption rates at different ethanol concentrations.

Figure 4A shows the adsorption rate of *Ziziphus jujuba* mesocarp extract at various pH levels. The adsorption rate was notably higher under alkaline conditions, peaking at pH 10, which was subsequently chosen for macroporous resin elution. As depicted in Figure 4B, the maximum adsorption capacity was reached when 40 mL of the sample with a 10% concentration approached the theoretical leakage point. At 80 mL, the resin reached its saturation point, and further addition of *Ziziphus jujuba* extract resulted in incomplete adsorption. Figure 4C shows the desorption rate across ethanol concentrations, where a gradual increase was observed as the ethanol concentration increased from 20% to 80%. The desorption rate peaked at 80% ethanol. Following the optimal extraction process, the extract was concentrated under reduced pressure to remove residual alcohol. The pH was adjusted to 10 before adsorption onto a pretreated D-101 macroporous resin column and eluted with 80% ethanol. The eluate was collected and concentrated. Total flavonoid content analysis revealed that the purity of TFZJM purified using D-101 macroporous resin reached 58.32%.

### 2.6. Analysis and Identification of Constituents in TFZJM

**Figure 5:**
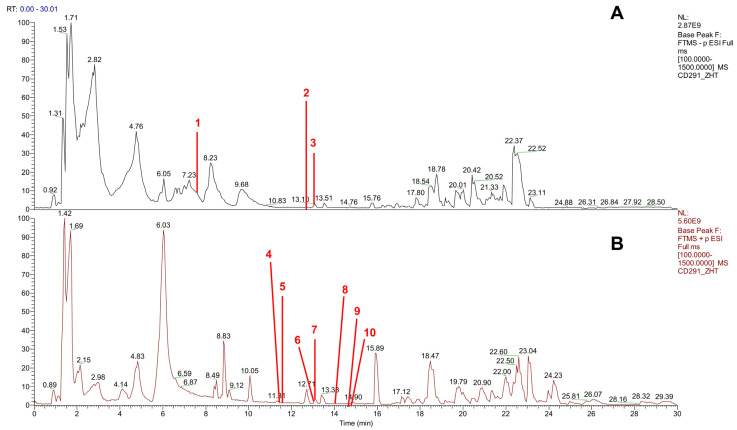
Total ion chromatograms in negative ion (**A**) and positive ion (**B**) modes.

**Figure 6:**
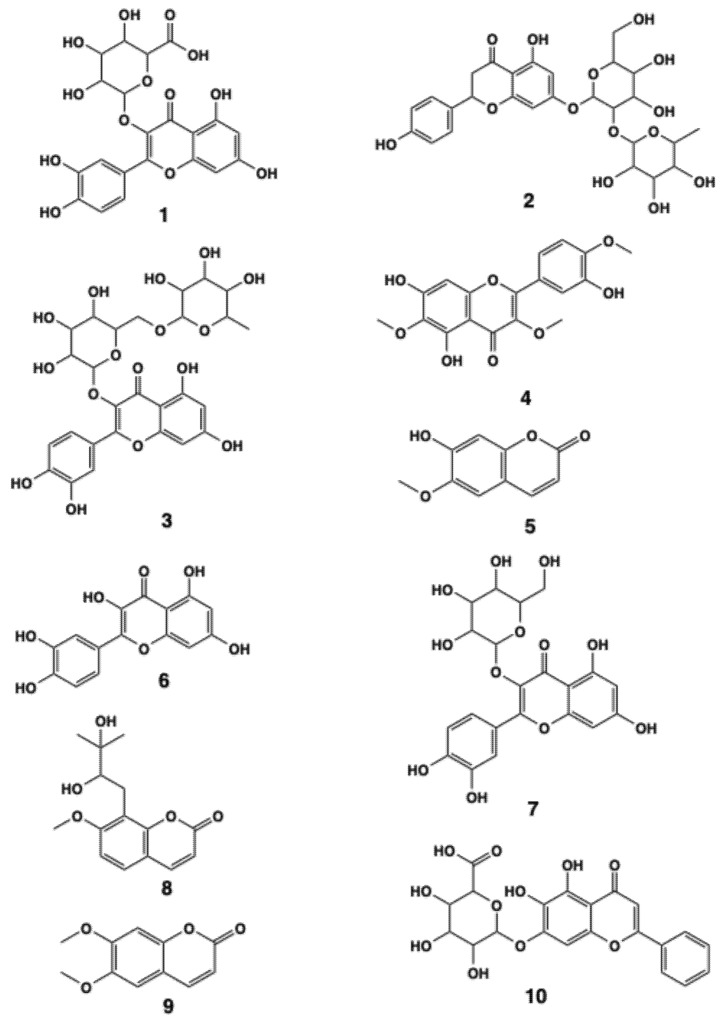
Chemical structural formula of the flavonoid components of TFZJM.

TFZJM was analyzed and compared using UPLC-Q-TOF-MS. The chromatogram is shown in Figure 5. Based on database comparisons, chromatographic peak retention time, and mass spectrometry fragmentation mode, a total of 10 flavonoid compounds were identified. The information and structure of the compounds are presented in Table 3 and Figure 6.

### 2.7. Sleep Latency and Sleep Duration After TFZJM Treatment

**Figure 7:**
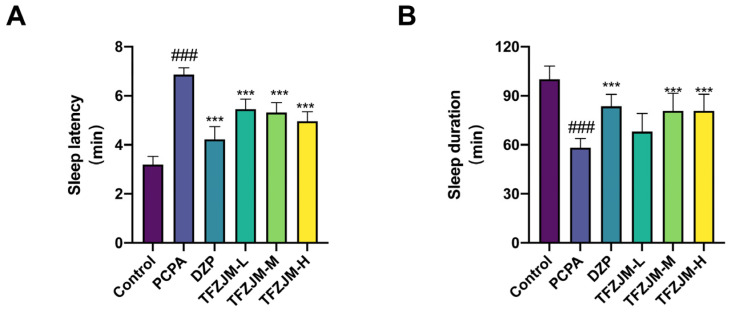
Effects of TFZJM on (**A**) sleep latency and (**B**) sleep duration in PCPA-induced insomnia model mice. Data are presented as mean ± SD. Comparisons with the control group show ### *p* < 0.001; comparisons with the PCPA group indicate *** *p* < 0.001.

Of the 60 mice, 10 were randomly assigned to the blank group, while the remaining 50 were allocated to the model, DZP, TFZJM-L, TFZJM-M, and TFZJM-H groups following successful insomnia model induction via an intraperitoneal injection of PCPA. Continuous gastric administration was performed in all groups. After 8 days of treatment, a sodium pentobarbital-induced sleep experiment was conducted, and sleep latency and duration were recorded for each group. As shown in Figure 7, the model group exhibited a significant increase in sleep latency compared to the blank group (*p* < 0.01). Sleep latency in the DZP and TFZJM dosage groups was significantly reduced compared to the model group (*p* < 0.01), with TFZJM-H showing the shortest latency among the TFZJM dosage groups, followed by TFZJM-M, with TFZJM-L displaying the longest. Figure 7 shows that the model group experienced a marked reduction in sleep duration relative to the blank group (*p* < 0.01). Sleep duration in the DZP, TFZJM-H, and TFZJM-M groups was significantly increased compared to that in the model group (*p* < 0.01), while the increase in the TFZJM-L group was not statistically significant. Among the TFZJM dosage groups, TFZJM-H resulted in the longest sleep duration, followed by TFZJM-M, and TFZJM-L resulted in the shortest sleep duration.

### 2.8. HE Staining Analysis

**Figure 8:**
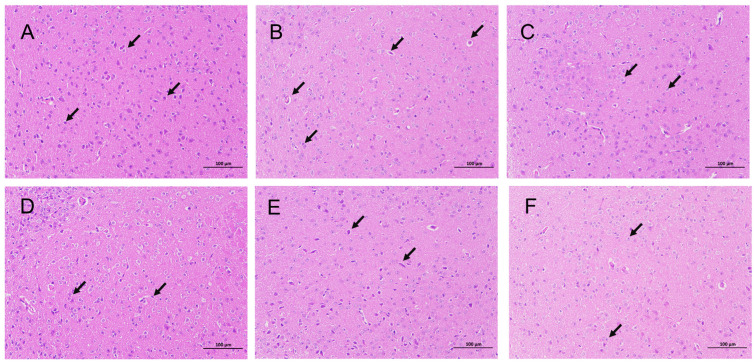
Effect of TFZJM on pathological morphology of mouse brain tissue (HE, magnification ×200). (**A**) Control; (**B**) model; (**C**) DZP; (**D**) TFZJM-H; (**E**) TFZJM-M; (**F**) TFZJM-L. Note: Black arrows indicate pathological changes in the nuclei of hypothalamus neurons in the brain tissue.

As depicted in Figure 8, HE staining revealed an irregular cellular arrangement in the brain tissue of the model group compared to the blank group. In the hypothalamus, numerous neurons exhibited shrunken, intensely stained nuclei, deformed cell bodies, and indistinct boundaries between the nuclei and cytoplasm. Additionally, extensive neuronal degeneration was observed, characterized by a loose and lightly stained cytoplasm. In contrast, brain tissues from the DZP and TFZJM treatment groups displayed clearer staining. Neuronal nuclei shrinkage was partially alleviated in the hypothalamus, with reduced signs of degeneration observed. These results indicate that TFZJM contributes to the improvement of brain tissue morphology in mice.

### 2.9. ELISA Results

**Figure 9:**
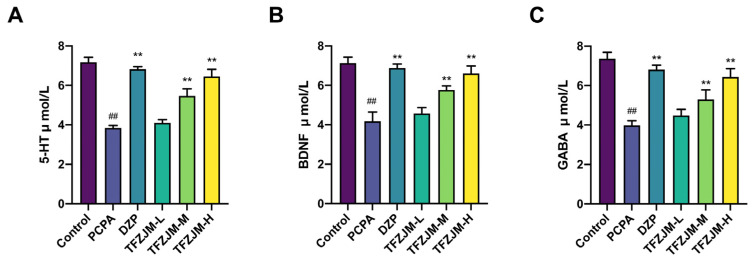
Measurement of (**A**) 5-HT, (**B**) BDNF, and (**C**) GABA levels in the brain tissue of mice in each group using ELISA. Data were presented as mean ± SD. Comparisons to the control group show, ## *p* < 0.01; comparisons to the PCPA group indicate, ** *p* < 0.01, n = 6.

ELISA analysis (Figure 9) revealed significant reductions in 5-HT, GABA, and BDNF levels in the model group compared to the blank group (*p* < 0.01). However, the administration of DZP, TFZJM-H, and TFZJM-M resulted in significant increases in 5-HT, GABA, and BDNF levels (*p* < 0.01) compared with the model group. In contrast, the TFZJM-L group did not show a statistically significant increase in any of these biomarkers. Among the TFZJM dosage groups, the 5-HT, GABA, and BDNF levels followed a descending order: TFZJM-H, TFZJM-M, and TFZJM-L

### 2.10. PCR Analysis

**Figure 10:**
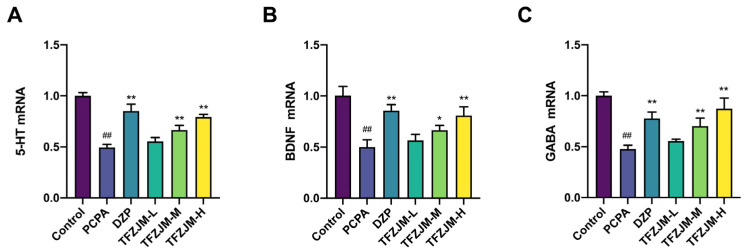
The mRNA expression levels of 5-HT1AR, BDNF, and GABAARα1 in each group of mice measured by PCR. (**A**) mRNA expression level of 5-HT1AR; (**B**) mRNA expression level of BDNF; (**C**) mRNA expression level of GABAARα1. Data were presented as mean ± SD. Comparisons to the control group show ## *p* < 0.01; comparisons to the PCPA group indicate * *p* < 0.05, ** *p* < 0.01, n = 3.

Analysis of the PCR results (Figure 10) revealed a significant reduction in the mRNA expression levels of 5-HT1AR, GABAARα1, and BDNF in the model group compared to the blank group (*p* < 0.01). In contrast, the mRNA expression levels of 5-HT1AR, GABAARα1, and BDNF in the DZP, TFZJM-H, and TFZJM-M groups were notably increased relative to those in the model group (*p* < 0.05). However, the increase in mRNA expression in the TFZJM-L group was not statistically significant. Among the different TFZJM dosage groups, the expression levels of 5-HT1AR, GABAARα1, and BDNF followed the order of TFZJM-H, TFZJM-M, and TFZJM-L.

### 2.11. Western Blotting Results

**Figure 11:**
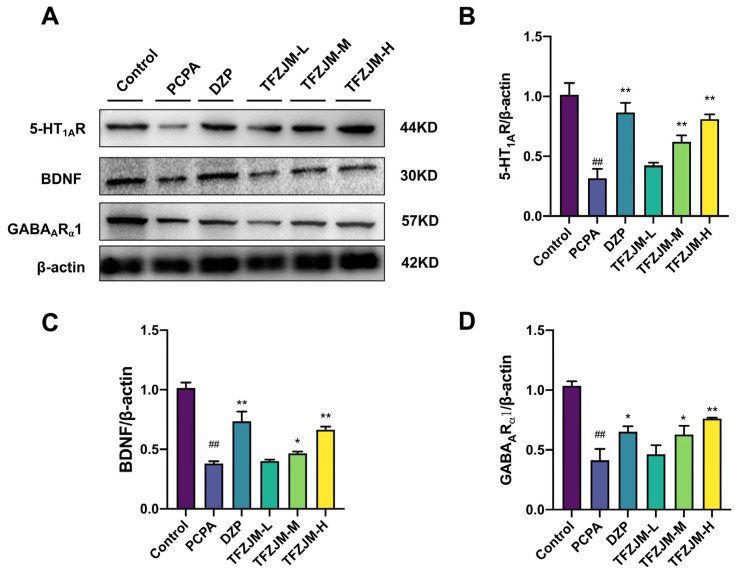
(**A**) Western blotting analysis of 5-HT1AR, BDNF, and GABAARα1 in mice of each group; (**B**) proportion of immunoblotting bands of 5-HT1AR, (**C**) BDNF to Beta relative to Beta Actin, and (**D**) GABAARα1. Data were presented as mean ± SD. Comparisons to the control group show ## *p* < 0.01; comparisons to the PCPA group indicate * *p* < 0.05, ** *p* < 0.01, n = 3.

As depicted in Figure 11, WB analysis revealed that the model group exhibited a significant reduction in the protein expression of 5-HT1AR, GABAARα1, and BDNF compared with the blank group (*p* < 0.01). Treatment with DZP, TFZJM-H, and TFZJM-M significantly upregulated the expression of 5-HT1AR, BDNF, and GABAARα1 compared to the model group (*p* < 0.05). These results suggest that both medium and high doses of TFZJM exert anti-insomnia effects, potentially through the modulation of 5-HT1AR, GABAARα1, and BDNF expression.

### 2.12. TFZJM Serum Metabolomics Analysis

**Figure 12:**
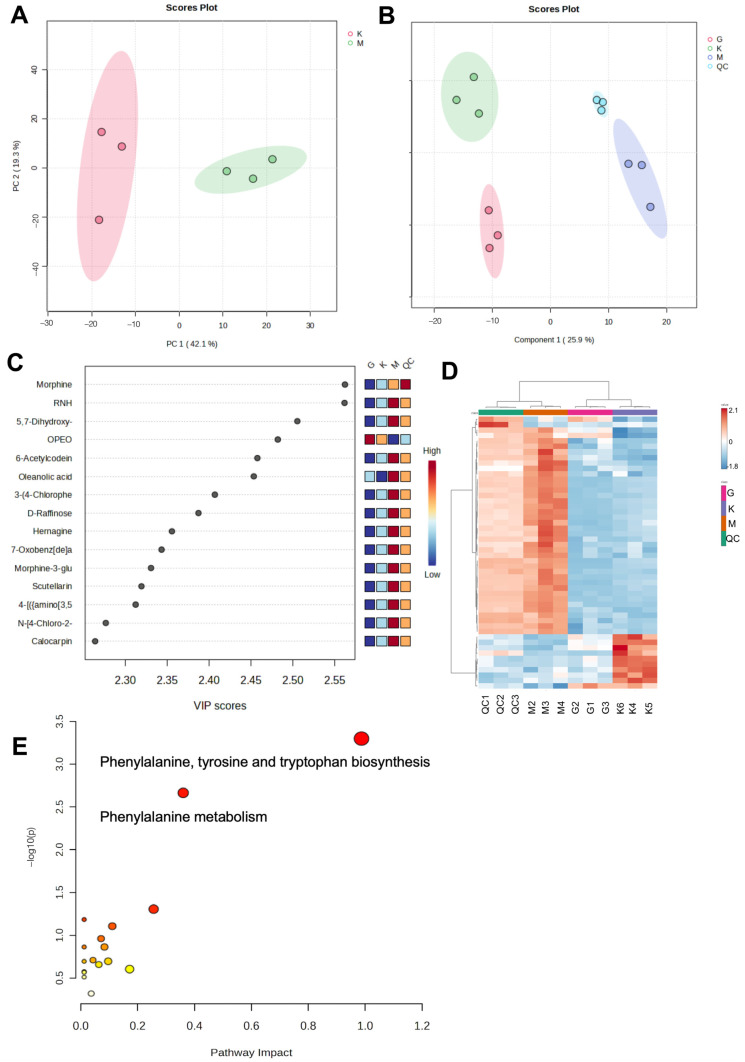
Effect of TFZJM on differential metabolite expression in PCPA–induced insomnia model mice. (**A**) PCA plot of the DEMs in each group; (**B**) PLS–DA in each group; (**C**) VIP results; (**D**) Heatmap of the DEMs in each group; (**E**) Metabolic pathway enrichment analysis diagram of the DEMs. Note: In the metabolic pathway enrichment bubble plot, the color of the bubbles represents the significance of enrichment: the color of the bubbles reflects the size of the *p*-value, and the redder the color, the larger −log10 (*p*-value), and the whiter the color, the smaller −log10 (*p*-value).

Metabolomics and multivariate statistical analysis based on UPLC-Q-TOF-MS were employed to assess alterations in serum metabolomics in mice following the administration of TFZJM. PCA of the non-targeted metabolomics profile revealed significant differences between the control and model groups (Figure 12A), confirming the successful establishment of the PCPA-induced insomnia mouse model. Comparative analysis across the control, model, and treatment groups using PCA (Figure 12B) and cluster heat map analysis (Figure 12D) demonstrated tight clustering within each group, indicating minimal intragroup variability. Furthermore, the proximity of metabolite clusters in the treatment group to those in the control group suggested normalization of the metabolic profile in PCPA-induced insomnia mice following administration. PLS analysis was used to generate VIP scores for each metabolite, identifying those most relevant to the separation of the two groups in both negative and positive ion modes (Figure 12C). To further elucidate the metabolic pathways affected by TFZJM, MetaboAnalyst 6.0 was used to analyze the significantly altered metabolites. Differentially expressed metabolites (DEMs) between the control and model groups, along with those reversed by flavonoid treatment, are listed in Table 4. The non-targeted metabolomics results (Figure 12E) indicated that the most affected pathways included phenylalanine, tyrosine, cytochrome P450, and alanine metabolism.

## 3. Discussion

Flavonoids present in *Ziziphus jujuba* seeds are known for their sedative and hypnotic effects. While previous studies have confirmed the presence of flavonoids in *the Ziziphus jujuba* mesocarp, research on TFZJM remains limited. *Ziziphus jujuba* mesocarp offers an abundant source of raw material for flavonoid extraction, though it also contains other compounds such as organic acids. The ethanol reflux extraction of TFZJM has the advantages of good solubility, higher purity of the target substance owing to impurity separation, a high extraction yield, and preservation of its related activity. In this study, the optimal extraction conditions were determined based on a single-factor experiment using the Box–Behnken method. The optimal extraction conditions were as follows: 60% ethanol concentration, SLR of 1:25 g·mL^−1^, and 60 min extraction time. The maximum yield of TFZJM was 1.98%. The extracted material obtained by optimizing the extraction process is a crude extract containing TFZJM, which needs to be purified and concentrated. After purification using the D101 macroporous resin, the purity reached 58.32%, indicating that the purification process has a higher purification efficiency and is more suitable for purifying TFZJM. This can be used for subsequent pharmacological activity research, indicating that the extraction process is simple, accurate, and feasible. An optimized extraction process is essential to maximize the retention of active flavonoid components, providing a solid theoretical foundation for the broader utilization of non-medicinal parts of *Ziziphus jujuba*.

At present, there are relatively few studies on the pharmacodynamic components of TFZJM for the prevention and treatment of insomnia. Many flavonoid components may serve as the pharmacodynamic basis for TFZJM. In this study, the flavonoid components in TFZJM were analyzed using UPLC-Q-TOF-MS, and 10 flavonoids, including miquelianin, naringin, rutin, quercetin, scoparone, and baicalin, were identified. Kim et al. found that miquelianin enhanced the quantity and quality of sleep through the GABAergic pathway [27]. Hernández-Vázquez et al. demonstrated that a single oral treatment rich in naringin extract produced a powerful anti-anxiety effect in mice [28]. Hernandez-Leon et al. discovered that rutin has an anti-anxiety effect, and the possible mechanism is related to the regulation of GABA_A_ receptors in the amygdala [29]. Bappi et al. demonstrated that quercetin exhibited dose-dependent antidepressant-like effects in thiopental sodium-induced mice [30]. Rebeca et al. demonstrated that when baicalin is directly injected into the central nervous system, it can promote anti-anxiety and sedative effects, and its pharmacological activity depends on the GABAergic non-benzodiazepine site [31]. Therefore, the analysis of these 10 flavonoid components lays the foundation for further research on the pharmacodynamic material basis of TFZJM.

Insomnia, a prevalent clinical condition, significantly disrupts the daily functioning of individuals. Despite extensive research, its pathogenesis remains unclear, and several hypotheses have been proposed to explain its development [32]. The leading theories include monoamine or its receptors, neuroendocrine, neuronal damage, and cellular and molecular mechanism hypotheses, with the “neurotransmitter hypothesis” being widely accepted among researchers [33,34]. Contemporary studies indicate that neurotransmitter balance directly influences sleep quality, as neurotransmitters mediate communication between neurons and are critical for brain function, particularly during sleep. Their release and regulation are directly linked to sleep quality [6,35]. Furthermore, research has identified a strong association between insomnia and the dysregulation of monoamine and amino acid neurotransmitters within the central nervous system, with significant reductions in 5-HT, GABA, and BDNF levels observed in insomniac mice [36]. 5-HT, a neurotransmitter integral to the central nervous system function, influences sleep, cognition, and various physiological processes. Reduced 5-HT levels have been implicated in conditions such as sleep disorders [37]. Liu et al. indicated that cannabidiol exerts sedative and hypnotic effects by increasing the content of 5-HT in the hypothalamus of PCPA-induced mice [12]. GABA, an inhibitory neurotransmitter, regulates neuronal excitability, maintains the balance of brain activity, and promotes relaxation and sleep. Diminished GABA levels are strongly correlated with sleep disorders and related symptoms [38]. Ye et al. found that cinnamic acid improved PCPA-induced insomnia by increasing the levels of 5-HT and GABA and inhibiting central excitability [39]. Investigating the role of these neurotransmitters in the brain is critical for understanding sleep regulation [40]. BDNF, a neurotrophic factor primarily active in the central nervous system, plays a fundamental role in neuroplasticity, mood regulation, and sleep [41]. Schmitt et al. investigated the serum BDNF levels of currently symptomatic adults with insomnia and non-sleep-disturbed control subjects, demonstrating that the serum BDNF levels of subjects currently experiencing insomnia symptoms were lower than those of the sleep-healthy control group and were significantly correlated with the severity of insomnia [42]. Wu et al. found that the alcoholic extract of Sophora flavescens improved PCPA-induced insomnia by promoting the transduction of PI3K/AKT/BDNF signals [43]. Alterations in BDNF expression are linked to the onset and progression of neurological conditions, making it essential to evaluate nervous system health and conditions such as insomnia and memory disorders [44,45]. Non-targeted metabolomics research on serum and brain biomarkers in insomnia model rats has identified phenylalanine and tryptophan metabolism as the most significantly impacted pathways associated with insomnia [46,47,48]. Consistent with the literature, our results indicate a close relationship between the pharmacological effects of TFZJM and neurotransmitters such as GABA, 5-HT, and BDNF.

The therapeutic efficacy of Chinese medicine extends beyond symptom relief and targets the underlying internal imbalances within the body. Metabolomic analysis revealed that PCPA significantly disrupted metabolites, including L-glutamate, L-tyrosine, and L-phenylalanine, in mouse serum. Notably, L-glutamate, a key biomarker of insomnia, plays a central role in glutamate metabolism, which is closely linked to the pathogenesis and progression of insomnia [49]. The present findings demonstrate that TFZJM exerts a substantial regulatory effect on the disruption of metabolites such as L-glutamate, L-tyrosine, and L-phenylalanine, indicating a potential therapeutic benefit for insomnia. Additionally, glutamate, as a precursor of GABA, is converted into GABA through the action of glutamate decarboxylase. Glutamate and GABA metabolism may reflect the equilibrium between neural excitation and inhibition [50]. A significant reduction in GABA levels further supported this finding, which is consistent with previous research. Serotonin (5-HT) is an indoleamine. The synthesis of serotonin begins with L-tryptophan, an essential amino acid obtained from dietary sources [51]. Agus et al. demonstrated that controlling the metabolic process of L-tryptophan could indirectly affect the glutamatergic pathway of the microbiome–gut-brain axis [52]. Studies have shown that serotonin is a sleep promoter, and blocking 5-HT synthesis can lead to long-term insomnia [53]. Furthermore, studies have found that in the brain, extracellular vesicles (EVs) of psychobiotics regulate the expression of 5-HT by upregulating brain-derived BDNF [51]. The neurotrophic function of BDNF is related to various physiological functions in the brain, especially neural plasticity, memory, and sleep [54,55,56,57]. Additionally, TFZJM regulates metabolic pathways related to phenylalanine, tyrosine, and alanine metabolism. L-Phenylalanine, an essential amino acid, is metabolized to tyrosine via phenylalanine hydroxylase, which is subsequently converted to dihydroxyphenylalanine by tyrosine hydroxylase. This process leads to the production of excitatory neurotransmitters, such as norepinephrine and dopamine, both of which are associated with insomnia [58,59]. In summary, TFZJM may exert therapeutic effects on insomnia by regulating metabolites such as L-glutamic acid, L-tyrosine, and L-phenylalanine, as well as the neurotransmitters derived from them.

Although this study provides valuable insights into the extraction of TFZJM and its sedative-hypnotic effects in a mouse model of insomnia, there are still some limitations. First, the number of animal samples used in this study was limited, which may have affected the statistical significance and generalizability of the results. Future studies should increase the sample size to enhance the reliability and accuracy of their results. Secondly, the study only used a PCPA-induced insomnia mouse model and did not involve other insomnia models or clinical research. Future research should consider using multiple insomnia models and clinical trials to verify the applicability of the results. Additionally, although this study explored the effects of TFZJM on certain neurotransmitters and metabolic pathways, the mechanism of action of TFZJM was not sufficiently explored. Future research should further investigate the specific mechanisms of action of TFZJM at the molecular and cellular levels. Finally, this study mainly focused on the short-term sedative-hypnotic effects and did not evaluate the long-term effects and safety of TFZJM. Future studies should include long-term administration experiments to assess the long-term efficacy and potential side effects.

## 4. Materials and Methods

### 4.1. Material

This study employed a variety of advanced instruments, including a 5810R high-speed refrigerated centrifuge (Eppendorf, Hamburg, Germany), KQ-200TDE CNC ultrasonic cleaner (Kunshan Ultrasonic Instruments Co., Ltd., Kunshan, Jiangsu, China), and 5430R high-speed centrifugal grinder (Eppendorf, Hamburg, Germany). Precision measurements were ensured using a Sartorius CPA225D 1/100,000 electronic balance (Beijing Sartorius Scientific Instrument Co., Ltd., Beijing, China). Quantitative and qualitative analyses were performed using a Thermo Multiskan GO multifunctional microplate reader (Thermo Fisher Scientific, Waltham, MA, USA) and a qTOW-ER2.2 real-time fluorescence quantitative PCR instrument (ANALYTIK JENA, Jena, Germany), respectively. Microscopic observations were conducted using an Eclipse Ci-L upright white light camera microscope (Nikon, Tokyo, Japan), and absorption spectra were recorded using a UV-2600 ultraviolet–visible spectrophotometer (Shimadzu Corporation, Kyoto, Japan). Methanol: Chromatographic grade, Thermo Fisher Scientific (China) Co., Ltd. (Shanghai, China); Formic acid: Chromatographic grade, Shanghai Aladdin Biochemical Technology Co., Ltd. (Shanghai, China); Pure water/ultrapure water integrated machine system: Direct-Q^®^5, Merck Millipore, Darmstadt, Germany; Fast micro refrigerated centrifuge: D3024R, Beijing Dalong Xingchuang Experimental Instrument Co., Ltd. (Beijing, China); Vortex oscillator: MX-F, Wuhan Seville Biotechnology Co., Ltd.; Ultrasonic cleaner: JP-040S, Shenzhen Jiemeng Cleaning Equipment Co., Ltd. (Shenzhen, China); Chromatograph: UltiMate 3000 RS, Thermo Fisher Scientific (China) Co., Ltd.; Mass spectrometer: Q Exactive high-resolution mass spectrometer, high-resolution mass spectrometer, Thermo Fisher Scientific (China) Co., Ltd.

Rutin (purity ≥ 98%, HR1713S1, Baoji Herbest Bio-Tech Co., Ltd., Baoji, China), anhydrous ethanol, sodium nitrite, sodium hydroxide (analytical grade, Tianjin Tianli Chemical Reagent Co., Ltd., Tianjin, China), and aluminum nitrate (analytical grade, Chengdu Chrom Chemicals Co., Ltd., Chengdu, China) were used in the experimental procedures. Additional reagents included PCPA (analytical grade, Sigma, Ronkonkoma, NY, USA), diazepam (2.5 mg/tablet, 220,306, Shandong Xinyi Pharmaceutical Co., Ltd., Dezhou, China), and antibodies such as 5-HT1AR (AC231209084, Wuhan Servicebio Technology Co., Ltd., Wuhan, China), GABAARα1 (AC231216033, Wuhan Servicebio Technology Co., Ltd., Wuhan, China), and BDNF (AC231209053, Wuhan Servicebio Technology Co., Ltd., Wuhan, China). ELISA kits for 5-HT (MM-0443M1), GABA (MM-0442M1), and BDNF (MM-0204M1) were sourced from Jiangsu Meimian Industrial Co., Ltd. (Yancheng, Jiangsu, China). RNA was extracted from brain tissue using the M5 HiPer Universal RNA Mini Kit (MF036, Mei5 Biotechnology, Beijing, China). For reverse transcription and quantitative PCR, the M5 Sprint qPCR RT kit with gDNA remover (MF166, Mei5 Biotechnology, Beijing, China) and 2X M5 HiPer SYBR Premix EsTaq kit (MF787, Mei5 Biotechnology, Beijing, China) were used. Staining was performed using hematoxylin (CR2311076, Servicebio, Wuhan, China) and eosin (CR2402037-5, Servicebio, Wuhan, China).

The *Ziziphus jujuba* sample, harvested in October 2022 from Hengshan District, Yulin, Shaanxi Province, was authenticated by Zhongxing Song, chief pharmacist at the Shaanxi Provincial- and Ministerial-Level Collaborative Innovation Center for Chinese Medicine Resources Industrialization. The specimen was confirmed to be the dried fruit of *Ziziphus jujuba* Mill. var. *spinosa* (Bunge) Hu ex H. F. Chou, a member of the Rhamnaceae family. The *Ziziphus jujuba* var. spinosa specimen was stored at the Institute of Medicinal Plant Development, Chinese Academy of Medical Sciences (specimen repository code: PE02040730).

### 4.2. Assay of Total Flavonoid Content

#### 4.2.1. Plotting of Standard Curve

The total flavonoid content was quantified using the NaNO_2_-Al(NO_3_)_3_-NaOH colorimetric method [60]. A rutin reference solution (0.2 mg·mL^−1^) was prepared by dissolving the reference material in 65% ethanol. Specific volumes of the reference solution (0.0, 2.0, 3.0, 4.0, 5.0, 6.0, and 7.0 mL) were accurately transferred to 25 mL volumetric flasks. Subsequently, 1.0 mL of 5% NaNO_2_ solution was added, mixed thoroughly, and allowed to stand for 6 min. Next, 1.0 mL of 10% Al(NO_3_)_3_ solution was added, followed by a 6 min resting period after mixing. Finally, 10.0 mL of 4% NaOH solution was added, and the flasks were filled to the mark with distilled water. The mixtures were shaken well and allowed to stand for 15 min. Absorbance was then measured at 510 nm. A standard curve was plotted using rutin concentration on the *x*-axis and absorbance on the *y*-axis, from which the regression equation was derived.

#### 4.2.2. Determination of Total Flavonoid Content in Sample Solution

By manually removing the *Ziziphus jujuba* pits from the dried *Ziziphus jujuba* mesocarp, the mesocarp is dried in a 40 °C oven, crushed, and then passed through a 50-mesh sieve until it reaches a constant weight, obtaining mesocarp powder. A precise 1 g powder was weighed and extracted under reflux with ethanol concentrations of 50%, 60%, 70%, 80%, and 90%; SLR of 1:15, 1:20, 1:25, 1:30, and 1:35 mg·mL^−1^; extraction times of 30, 60, 90, 120, and 150 min; and extraction frequencies of 1, 2, 3, 4, and 5. The influence of varying ethanol concentrations, SLR, extraction time, and extraction frequency on flavonoid extraction from the mesocarp was systematically evaluated. The total flavonoid level was quantified using a linear equation derived from the standard curve, and the extraction efficiency was computed using the following formula:

Total flavonoid extraction efficiency = (C × V × D)/m × 100%

where C represents the total flavonoid concentration (mg·mL^−1^), determined from the standard curve; V denotes the extract volume (mL); D is the dilution factor; and m is the sample mass (mg).

#### 4.2.3. Single-Factor Experiment for Extraction Process

Single-factor experiments were conducted according to the conditions in Section 4.2.2 to investigate the effects of ethanol concentration, SLR, extraction time, and extraction frequency on the yield of total flavonoids. Each parameter was tested in triplicate for consistency.

#### 4.2.4. Response Surface Optimization and Design of Extraction Process

Following the single-factor experiments, a response surface design was applied using the experimental matrix outlined in Table 5. The extraction efficiency of total flavonoids (Y, %) was used as the response variable. Verification experiments were subsequently performed based on the predicted optimal extraction parameters to determine the conditions that maximized flavonoid extraction efficiency.

### 4.3. UPLC-Q-TOF-MS Analysis

#### 4.3.1. Sample Preparation

After purification, the extract was freeze-dried for LC-MS/MS analysis. A 0.1 g freeze-dried powder was weighed, and 1 mL of 80% methanol and grinding beads were added, followed by grinding for 5 min. The mixture is vortexed for 10 min to ensure homogeneity, centrifuged at 13,000 rpm for 10 min, and the supernatant is collected for machine analysis.

#### 4.3.2. Mass Spectrometry Conditions

Ion source: electrospray ionization source (ESI) Scanning mode: Positive and negative ion switching scanning; Detection method: full mass/dd-MS2; Resolution: 70,000 (full mass) 17,500 (dd-MS2); scan range: 100.0–1500.0 *m*/*z*; Electrospray Voltage (Spary Voltage): 3.2 kV (Positive, Negative); Capillary Temperature: 300 °C; Collision gas: high-purity argon (purity ≥ 99.999); Collision energy (N)CE: 30, 40, 60; Sheath gas: nitrogen (purity ≥ 99.999), 40 Arb; Auxiliary gas: nitrogen (purity ≥ 15 Arb, 350 °C); Data collection time: 30.0 min.

#### 4.3.3. Chromatographic Conditions

Chromatographic column: AQ-C18, 150 × 2.1 mm, 1.8 μm, Welch; Flow rate: 0.30 mL/min Aqueous phase: 0.1% formic acid aqueous solution Organic phase: methanol Column oven temperature: 35 °C; Autosampler temperature: 10.0 °C; The injection volume of the autosampler is 5.00 μL.

#### 4.3.4. Data Analysis

The data collected by high-resolution liquid chromatography-mass spectrometry were retrieved and compared using CD 3.3 (Compound Discoverer 3.3). After the initial data organization was completed by Thermo Fisher, the data were retrieved and compared in the database mzCloud.

### 4.4. Drug Preparation

#### 4.4.1. Preparation of PCPA Suspension

A 0.3% CMC-Na solution was prepared using physiological saline and heated in a water bath with magnetic stirring at 70 °C and 600 r·min^−1^. CMC-Na was gradually added until fully dissolved. Subsequently, 12 g of PCPA powder was weighed, mixed with the CMC-Na solution, and diluted to a total volume of 300 mL. The suspension had a final concentration of (40 g·L^−1^) [61].

#### 4.4.2. Preparation of TFZJM Intragastric Solution

*Ziziphus jujuba* mesocarp was dried, powdered, and subjected to ethanol reflux extraction under optimized conditions: 60% ethanol concentration, an SLR of 1:25 g·mL^−1^, and an extraction time of 60 min. The resulting extract was concentrated under reduced pressure to eliminate residual ethanol. Enrichment and purification were performed using D-101 macroporous resins. After loading 80 mL of the sample solution onto a preconditioned D-101 resin column, the pH was adjusted to 10. The sample was then eluted with 80% ethanol, and the eluate was concentrated and freeze-dried to yield TFZJM powder. The powder was dissolved in distilled water and stirred to prepare an intragastric TFZJM solution.

#### 4.4.3. Preparation of Diazepam Intragastric Solution

Four diazepam tablets (10 mg per tablet) were pulverized into a fine powder, and purified water was added to reach a final volume of 50 mL, yielding a concentration of 0.1 mg·mL^−1^.

### 4.5. Animals

A total of 60 SPF male KM mice (40 ± 2 g) were obtained from Chengdu Dossy Experimental Animals Co., Ltd., Chengdu, China (Certificate number: SCXK (Chuan) 2020-0030). The mice were kept under controlled conditions at 23 ± 1.5 °C and 50–60% relative humidity, with no restriction on food or water, and maintained on a 12 h light/dark cycle. The experimental procedures were approved by the Ethics Committee of the Shaanxi University of Chinese Medicine (approval number: SUCMDL20230913001).

### 4.6. Animal Modeling and Administration

Male KM mice (n = 60) were randomly assigned to six groups, with 10 mice per group: (1) control, (2) model, (3) diazepam (DZP), (4) TFZJM-L, (5) TFZJM-M, and (6) TFZJM-H groups. The model was induced by intraperitoneal injection of PCPA at a dosage of 400 mg·kg^−1^ per day for four consecutive days [61]. During the modeling phase, all mice, except those in the control group, progressively exhibited behaviors such as mania, biting, increased aggression, disrupted circadian rhythms, dry fur, and decreased food consumption, confirming the successful establishment of the model. Subsequently, the drug was administered via gavage once daily for 8 days. Mice in both the control and model groups received pure water, while the other groups received the corresponding drugs: DZP (1.3 mg·kg^−1^), TFZJM-L (27 mg·kg^−1^), TFZJM-M (54 mg·kg^−1^), and TFZJM-H (108 mg·kg^−1^). After completing the sodium pentobarbital potentiation sleep experiment on the 8th day of treatment, the mice were euthanized via decapitation, and the brain tissues were immediately collected. Some tissues were preserved in fixative for later use, while others were stored at −80 °C for later testing.

### 4.7. HE Staining

Brain tissue was fixed for 48 h before embedding. Coronal sections (3 μm thick) were prepared, dried at 45 °C for 1 h, and then subjected to dewaxing and dehydration. Hematoxylin staining was performed, followed by differentiation using ethanol. After rinsing, the sections were treated with 0.6% ammonia water for bluing, rinsed again, and subsequently stained with eosin. The samples were then dehydrated, cleared, and mounted using neutral gum. Morphological observations were performed using a microscope at 200× magnification.

### 4.8. ELISA Analysis

Brain tissue samples were homogenized, followed by centrifugation at 4 °C and 12,000 r·min^−1^ for 10 min (centrifugal radius: 9.5 cm). The supernatant was collected, and an ELISA was performed according to the manufacturer’s protocol. The ELISA used in this experiment measured the 5-HT, GABA, and BDNF levels in the samples using a double-antibody sandwich method. Capture antibodies for purified mouse 5-HT, GABA, and BDNF were coated onto microplates to form solid-phase antibodies. The microplates were then incubated with the sample, followed by the addition of HRP-labeled detection antibodies. An antibody-antigen-enzyme-labeled antibody complex is formed and thoroughly washed. TMB is added to the wells, which turns blue in the presence of HRP enzyme and yellow in the presence of acid. The intensity of the color was directly proportional to the concentrations of 5-HT, GABA, and BDNF in the sample. The standard concentrations are 0, 0.5, 1, 2, 4, and 8 μmol/L. Each sample and standard is repeated three times. The absorbance at 450 nm (OD value) is measured using an ELISA reader. The minimum detectable sensitivity of the 5-HT, GABA, and BDNF ELISA kit was less than 0.1 μmol/L. We used the standard concentrations and OD values to calculate the linear regression equation for the standard curve (R^2^ = 0.99). The OD values of the samples were then input into the equation to calculate the sample concentrations, which were multiplied by the dilution factor to obtain the actual concentrations. The levels of 5-HT, GABA, and BDNF in different animal groups were compared. Statistical analysis was performed using a *t*-test, with *p* < 0.05 indicating statistical significance.

### 4.9. Western Blotting Analysis

Total protein from brain tissue was extracted using RIPA lysis buffer, and the protein concentration was quantified using a BCA protein assay kit (Borst Biotechnology Co., Ltd., Wuhan, China). Proteins were resolved on 8–12% SDS-polyacrylamide gel and transferred onto PVDF membranes. Membranes were blocked with 5% skim milk for 2 h and then incubated overnight at 4 °C with primary antibodies: 5-HT1AR (1:500), GABAARα1 (1:800), and BDNF (1:500). After three washes, membranes were incubated with HRP-related secondary antibodies (1:1000 dilution) for 2 h. Visualization was performed using an enhanced chemiluminescence kit. Statistical analysis was conducted using the *t*-test, with significance defined as *p* < 0.05.

### 4.10. RT-qPCR Analysis

Total RNA was extracted from brain tissue using the M5 HiPer Universal RNA Mini Kit (MF036, Mei5 Biotechnology, Beijing, China), followed by reverse transcription to cDNA using the M5 Sprint qPCR RT Kit (MF166, Mei5 Biotechnology, Beijing, China) with gDNA removal. RT-qPCR was subsequently conducted using the 2× M5 HiPer SYBR Premix EsTaq Kit (MF787, Mei5 Biotechnology, Beijing, China). The 2^−∆∆Ct^ method (CT value comparison method) was used for relative quantitative analyses. The relative expression value obtained by inputting the average Ct value of the target gene (such as 5-HT1AR, GABAARα1, and BDNF) and the corresponding housekeeping gene (such as GAPDH) into the formula is the relative expression level. The formula is as follows: F = 2^−[[the average Ct value of the target gene in the test group − the average Ct value of the housekeeping gene in the test group] − [the average Ct value of the target gene in the control group − the average Ct value of the housekeeping gene in the control group]]^. Each sample is repeated 3 times, and GAPDH, an internal reference gene, is added as a control to ensure the reliability and accuracy of the experimental results. The conditions for denaturation, annealing, and elongation during the PCR stage are as follows: 94 °C for 30 s, 55 °C for 30 s, and 72 °C for 30 s, for a total of 35 cycles. Primers for 5-HT1AR, GABAARα1, BDNF, and GAPDH were synthesized by Servicebio Co., Ltd. (Table 6).

### 4.11. Serum Metabolomics Analysis of TFZJM

#### 4.11.1. Serum Sample Processing

For serum sample preparation, 50 µL of frozen serum was mixed with 150 µL of pre-cooled methanol and vortexed for 10 min. The mixture was then centrifuged at 10,000 r·min^−1^ for 10 min at 4 °C, with a centrifugal radius of 9.5 cm. The supernatant was used for further analysis.

#### 4.11.2. Chromatography Mass Spectrometry Conditions

Mass spectrometry analysis and serum metabolite identification were conducted using an UltiMate 3000 RS chromatograph coupled with a Q Exactive high-resolution mass spectrometer (Thermo Fisher Scientific, Shanghai, China). Chromatographic separation was performed using an AQ-C18 column (150 mm × 2.1 mm, 1.8 μm) with a mobile phase consisting of 0.1% formic acid (A) and methanol (B). The gradient elution profile was as follows: 5–55% B from 0 to 5 min, 55–65% B from 5 to 9 min, 65–85% B from 9 to 14 min, 85–90% B from 14 to 20 min, 90–95% B from 20 to 24 min, 95% B from 24 to 28 min, 95–5% B from 28 to 29 min, and 5% B from 29 to 30 min. The flow rate was 0.3 mL·min^−1^, the volume was 5 μL, and the column temperature was set to 35 °C. Mass spectrometric detection was performed in both ion modes, with a scanning range of *m*/*z* 100–1500. High-purity nitrogen was supplied as the auxiliary spray ionization and desolvation gas, and nitrogen also served as the nebulizer and collision gas. The key parameters included a nebulizer temperature of 350 °C, a nebulizer gas flow rate of 8 L·min^−1^, a sheath gas temperature of 350 °C, and a sheath gas flow rate of 11 L·min^−1^. The capillary voltage was set to 3500 V for both ion modes, while nozzle voltages were adjusted to 1000 V for negative mode and 300 V for positive mode, with a pyrolysis voltage of 130 V.

### 4.12. Data Processing and Analysis

Chromatographic data from non-targeted metabolomics samples were acquired and processed using Xcalibur 4.1 (Thermo Fisher Scientific), with preliminary data sorting performed using CD3.3 (Thermo Fisher Scientific, Shanghai, China). Metabolite identification was performed by comparing the results with the mzCloud database. The processed data were further analyzed using PCA and OPLS-DA with SIMCA-P 14.1 software. Differential metabolites were screened using a VIP threshold of >1 and a significance level of *p* < 0.05. Potential biomarkers were subsequently confirmed through integration with the HMDB (https://hmdb.ca, accessed on 5 November 2023). Identified endogenous metabolites were mapped to relevant metabolic pathways using MetaboAnalyst 5.0, with an impact > 0.1 as the criterion for pathway selection.

## 5. Conclusions

This study employed response surface methodology with a three-variable, three-level central composite design to optimize the ethanol reflux extraction process of TFZJM. The resulting extraction method offers several advantages, including high cost-effectiveness, elevated extraction yield, and ease of operation. UPLC-Q-TOF-MS analysis identified 10 flavonoid compounds, including miquelianin, naringin, rutin, and quercetin. These compounds may play an important role in controlling the pharmacological characteristics of PCPA-induced insomnia. This study lays the foundation for further research on the pharmacodynamic substances of TFZJM. Furthermore, this study investigated the sleep-enhancing effects of TFZJM in PCPA mice by establishing a PCPA mouse model of insomnia. TFZJM-M and TFZJM-H were effective in improving insomnia in mice. Metabolomic analyses revealed that TFZJM can ameliorate insomnia by modulating metabolites such as L-glutamate, L-tyrosine, and L-phenylalanine, as well as metabolic pathways, including phenylalanine, tyrosine, and alanine metabolism. This lays the foundation for further investigation into the mechanisms through which TFZJM enhances learning and memory in PCPA mice. Therefore, this study provides valuable insights for the future development and utilization of *Ziziphus jujuba* wastewater, with significant implications for maximizing its resource value.

## Figures and Tables

**Table 1:** Box–Behnken experimental design and results for TFZJM extraction conditions.

Number	Extraction Time (A, min)	Ethanol Concentration (B, %)	Solid-to-Liquid Ratio (C, g·mL^−1^)	Total Flavonoid Yield (%)
1	60	70	20	1.58
2	60	60	25	1.89
3	90	60	30	1.37
4	60	70	30	1.67
5	90	60	20	1.49
6	60	60	25	1.97
7	60	50	20	1.54
8	60	60	25	1.91
9	30	60	30	1.55
10	30	70	25	1.42
11	90	50	25	1.35
12	60	60	25	1.95
13	60	60	25	1.88
14	30	60	20	1.32
15	90	70	25	1.39
16	60	50	30	1.56
17	30	50	25	1.35

**Table 2:** Analysis of variance results.

	Sum of Squares	Mean Square	*F* Value	*p* Value
Model	0.8696	0.0966	108.22	<0.0001
A	0.0002	0.0002	0.224	0.6504
B	0.0084	0.0084	9.46	0.0179
C	0.006	0.006	6.78	0.0353
AB	0.0002	0.0002	0.252	0.6311
AC	0.0306	0.0306	34.3	0.0006
BC	0.0012	0.0012	1.37	0.2798
A^2^	0.5121	0.5121	573.57	<0.0001
B^2^	0.1581	0.1581	177.03	<0.0001
C^2^	0.0811	0.0811	90.79	<0.0001
Residual error	0.0063	0.0009		
Lack of fit	0.0003	0.0001	0.0556	0.9805
Pure error	0.006	0.0015		
R^2^	0.9929			
R^2^_adj_	0.9837			
CV	1.87			

**Table 3:** Identification of TFZJM components.

No.	RT (min)	M/Z (Da)	Molecular Formula	Compound Name	Mode	CAS	Secondary Fragment
**1**	7.58	477.0700	C_21_H_18_O_13_	Miquelianin	[M-H]-1	22688-79-5	
**2**	12.67	579.1725	C_27_H_32_O_14_	Naringin	[M-H]-1	10236-47-2	583.13; 583.17; 583.19; 582.18; 581.18
**3**	13.09	609.1462	C_27_H_30_O_16_	Rutin	[M-H]-1	153-18-4	1219.3; 976.3; 975.3; 955.2; 783.1
**4**	11.49	361.0917	C_18_H_16_O_8_	5,7-dihydroxy-2-(3-hydroxy-4-methoxyphenyl)-3,6-dimethoxy-4*H*-chromen-4-one	[M+H]+1	17313-52-9	
**5**	11.61	193.0500	C_10_H_8_O_4_	7-hydroxy-6-methoxy-2*H*-chromen-2-one	[M+H]+1	92-61-5	197.12; 197.08; 196.1; 196
**6**	13.09	303.0497	C_15_H_10_O_7_	Quercetin	[M+H]+1	117-39-5	307.12; 307.14; 306.13; 306.1; 305.1
**7**	13.10	465.1028	C_21_H_20_O_12_	Quercetin-3β-D-glucoside	[M+H]+1	482-35-9	469.11; 468.13; 468.19; 4657.15; 466.17; 466.11
**8**	14.03	261.1125	C_15_H_18_O_5_	8-(2,3-dihydroxy-3-methylbutyl)-7-methoxy-2*H*-chromen-2-one	[M+H]+1	5673-37-0	263.1; 262.1; 261.08; 261.11; 258.11
**9**	14.65	207.0654	C_11_H_10_O_4_	Scoparone	[M+H]+1	120-08-1	233.08; 229.14; 229.15; 2226.11; 225.15
**10**	14.72	447.0924	C_21_H_18_O_11_	Baicalin	[M+H]+1	21967-41-9	5686.35; 91.82; 569.26; 522.18

**Table 4:** Comparison of DEM in the control; Model, and TFZJM administration group.

Number	Metabolite	HMDB	Tendency	
			Control Group vs. Model Group	Drug Group vs. Model Group
1	L-Tyrosine	HMDB0000158	↓ ^3)^	↑ ^3)^
2	L-Dopa	HMDB0000181	↓ ^3)^	↑ ^3)^
3	L-Isoleucine	HMDB0000172	↓ ^3)^	↑ ^3)^
4	L-Phenylalanine	HMDB0000159	↓ ^3)^	↑ ^3)^
5	trans-Aconitic acid	HMDB0000958	↑ ^3)^	↓ ^3)^
6	Oleanolic acid	HMDB0002364	↑ ^3)^	↓ ^3)^
7	D-Raffinose	HMDB0003213	↑ ^3)^	↓ ^3)^
8	Morphine	HMDB0014440	↑ ^3)^	↓ ^3)^
9	L-Glutamic acid	HMDB0000148	↓ ^3)^	↑ ^3)^
10	Decanoylcarnitine	HMDB0000651	↓ ^3)^	↑ ^3)^

Note: ^3^**^)^** *p* < 0.05; ↓: downregulated, ↑: upregulated.

**Table 5:** Factors and levels for response surface design.

Level	Factor		
	Extraction Time (A, min)	Ethanol Concentration (B, %)	Solid-to-Liquid Ratio (C, g·mL^−1^)
−1	30	30	1:20
0	60	60	1∶25
1	90	90	1∶30

**Table 6:** Primers sequence.

Primer Name	Segment Length	Forward Primer	Reverse Primer
BDNF	136	GCCCATGAAAGAAGTAAACGTCC	AGTGTCAGCCAGTGATGTCGTC
5-HT_1A_R	302	ACTCCACTTTCGGCGCTTTC	GGCTGACCATTCAGGCTCTTC
GABA_A_R_α_1	166	CCAAGTCTCCTTCTGGCTCAAC	CTTTTCTGGAACCACGCTTTTG
GAPDH	133	CCTCGTCCCGTAGACAAAATG	TGAGGTCAATGAAGGGGTCGT

## Data Availability

Data are contained within the article.

## Abbreviations

γ-aminobutyric acid (GABA), 5-hydroxytryptamine (5-HT), Analysis of Variance (ANOVA), brain-derived neurotrophic factor (BDNF), coefficient of variation (CV), diazepam (DZP), Differentially expressed metabolites (DEMs), para-chlorophenylalanine (PCPA), solid–liquid ratio (SLR), total flavonoids from *Ziziphus jujuba* mesocarp (TFZJM), Ultra-Performance Liquid Chromatography-Quadrupole-Time of Flight-Mass Spectrometry (UPLC-Q-TOF-MS), norepinephrine (NE), lateral hypothalamus (LHA), locus coeruleus (LC), proto-oncogene protein (C-Fos), glutamic acid (GLU).
